# Synthesis of polyaniline-sulfur composites with different nanostructures *via* an interfacial emulsification method and a micelle template method and their properties

**DOI:** 10.1039/d0ra00122h

**Published:** 2020-03-20

**Authors:** Jing Wang, Shichao Zhang

**Affiliations:** School of Material Science and Engineering, Bei Hang University China csc@buaa.edu.cn

## Abstract

Herein, polyaniline (PANI)-S with a nano-microsphere structure was synthesized *via* an interfacial emulsification method using Triton X-100 as an emulsifier and hydrochloric acid (HCl) as a dopant and covering element sulfur. Moreover, PANI-S with a nanotubular structure was synthesized *via* a micelle template method using sodium dodecyl sulfonate (SDS) as a template and HCl as a dopant, followed by heating with 40 wt%, 60 wt%, and 70 wt% element sulfur. The two kinds of polyaniline-sulfur (PANI-S) composites were separately characterized by scanning electron microscopy (SEM), FTIR spectroscopy, energy dispersive X-ray spectroscopy (EDS), thermogravimetric analysis (TGA), X-ray photoelectron spectroscopy (XPS), *etc.* Their electrochemical performances were also investigated, and the results showed that as the sulfur content increased, the electrochemical performance of the PANI-S (synthesized *via* the SDS/HCl template method) electrode improved due to the increase in the amount of the active substance in the electrode. Compared with nano-microsphere PANI-S, nanotube PANI-S has higher specific capacity (1102.01 mA h g^−1^), more stable cycle performance, and better rate performance, suggesting that an excellent lithium–sulfur battery can be prepared by designing an electrode material structure using nanotube PANI-S.

## Abstract 2

Herein, polyaniline (PANI)-S with a nano-microsphere structure was synthesized *via* an interfacial emulsification method using Triton X-100 as an emulsifier and hydrochloric acid (HCl) as a dopant and covering element sulfur.

## Graphical abstract

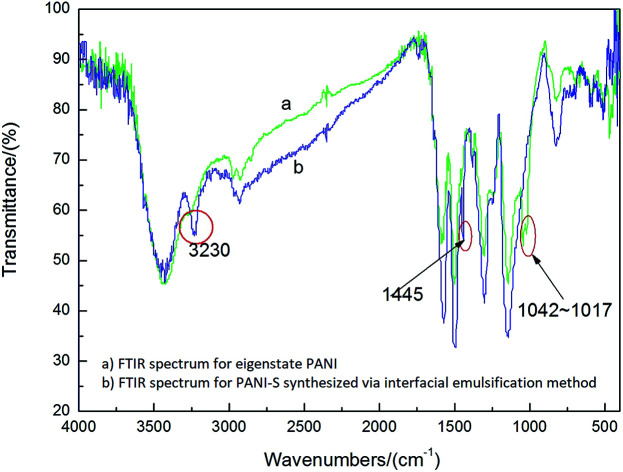

## 1. Introduction

The performance of a cathode material directly determines the properties of a lithium-ion battery.^1^ When the capacity of the positive electrode is increased by 100%, the total capacity of the lithium-ion battery will be increased by 68%; however, when the capacity of the negative electrode is increased by 100%, the total capacity of the lithium-ion battery will be increased by only 12%.^2^ Therefore, it has become important to develop appropriate cathode materials to improve the performance of lithium-ion batteries and reduce their cost effectively.^3^ The charge–discharge process of a lithium–sulfur battery involves a multielectron reaction, which will encourage higher theoretical energy density. Thus, if sulfur composites as an active positive electrode material can be properly modified or designed, the disadvantages (such as double insulated nature from electrons and ions and low utilization rate of sulfur active materials) of element sulfur can be offset; then, the high electrochemical capacity of sulfur can be fully utilized and the cycle performance of lithium-ion batteries will be further improved.^4–6^

Polyaniline is a kind of conducting polymer material containing alternating benzene rings and nitrogen atoms on its main chains. Polyaniline with a nanostructure has the advantages of high and adjustable conductivity, a unique doping mechanism, excellent physical properties, good environmental stability, inexpensive raw materials, simple synthesis method, *etc.*;^7,8^ moreover, it exhibits good oxidation–reduction reversibility during the charge–discharge process.^9^ Therefore, it has a great application value for advanced batteries. In 2011, Wu^10^*et al.* prepared a kind of composite with PANI as a shell and MWCNT-S as a core. The initial discharge capacity of this composite electrode reached 1334 mA h g^−1^ with the capacity retention rate of 70% after 80 cycles. Gao^11^*et al.* prepared a kind of water-dispersed PANI with a nanowire structure *via* heterogeneous nucleation. The initial discharge specific capacity of this composite electrode reached 927 mA h g^−1^ with the capacity retention rate of 88.3% after 100 cycles. In addition, An^12^*et al.* designed a kind of PANI-S-PANI composite with a hollow structure, which could effectively prohibit the volume expansion and shuttle effect during the charge–discharge process. The discharge specific capacity of this composite electrode reached 572.2 mA h g^−1^ with the coulombic efficiency of 87% after 214 cycles.

There are many common methods, such as template,^13^ emulsion polymerization,^14^ and electrochemical polymerization,^15^ for the preparation of nano-structure polyaniline. Different polymerization methods will have different influences on the structure and physical and chemical properties of polyaniline, which will then affect the morphologies and properties of the product.^16–18^

In this study, nano-microsphere polyaniline was synthesized *via* the interfacial emulsification method using Triton X-100 as an emulsifier and hydrochloric acid (HCl) as a dopant and covering element sulfur. Moreover, PANI with a nanotubular structure was synthesized *via* the micelle template method using sodium dodecyl sulfonate (SDS) as a template and HCl as a dopant, followed by heating with 40 wt%, 60 wt%, and 70 wt% element sulfur. Both structures of PANI-S were characterized by scanning electron microscopy (SEM), FTIR spectroscopy, thermogravimetric analysis (TGA), X-ray photoelectron spectroscopy (XPS), *etc.* Their electrochemical performances were compared, and the results showed that as the sulfur content increased, the electrochemical performance of PANI-S (synthesized *via* the SDS/HCl template method) improved due to the increase in the amount of the active substance in the electrode. Compared with nano-microsphere PANI-S, nanotube PANI-S had a higher specific capacity of up to 1102.01 mA h g^−1^, more stable cycle performance, and better rate performance, suggesting that an excellent lithium–sulfur battery can be prepared by designing an electrode material structure using nanotube PANI-S.

## 2. Experimental

### 2.1 Material preparation

At first, sulfur (AR, Beijing Yili Fine Chemicals Co., Ltd.) was dissolved in CS_2_ (Tianjin Baishi Chemical Industry Co., Ltd.), and then, 0.2 mol L^−1^ hydrochloric acid (HCl, AR) and Triton-X100 (octylphenol ethoxylate, Jiangsu Hai'an petrochemical plant) were added to this mixture solution. A certain amount of deionized water, aniline (Ji'nan Tian Shi Hao Trading Co., Ltd.), and ammonium persulfate (APS, AR, Beijing Yili Fine Chemicals Co., Ltd.) (1 : 1 mol feed ratios) was subsequently added followed by stirring for 24 hours. The final precipitate was washed, dried, and ground into powder.

A certain amount (as shown in Table 2) of sodium dialkyl sulfonate (SDS, AR, Beijing Yili Fine Chemicals Co., Ltd.) was dissolved completely in a HCl solution (0.2 mol L^−1^) and 350 mL deionized water; a certain amount of aniline was subsequently added, and the mixed solution was dispersed by ultrasonication for 1 hour. Then, ammonium persulfate (APS, 1 : 1 mol feed ratios with aniline) was slowly added, and the mixed solution was stirred for 24 hours at room temperature. The final precipitate was washed, dried, and ground into powder. Then, the product was mixed with different proportions of sulfur (0, 40 wt%, 60 wt% and 70 wt%) followed by heating at 160 °C for 24 hours.

### 2.2 Characterization

Both the PANI-S composite synthesized *via* the interfacial emulsification method and the PANI-S composite synthesized *via* the SDS/HCl template method were further characterized by infrared spectroscopy (IR, Nicolet-60SXB), energy dispersive X-ray spectroscopy (EDS, Apollo XLT), and X-ray photoelectron spectroscopy (XPS, Thermo Escalab 250Xi) to determine the elemental composition. The product was subjected to thermogravimetric analysis (TGA, TA-Q50) at the heating rate of 10°C min^−1^ to confirm the sulfur content. Scanning electron microscopy (SEM, Zooma 200, The Netherlands) and transmission electron microscopy (TEM, Tecnai G2 F30) were used to determine the topography of the materials.

### 2.3 Electrochemical measurement

Both the PANI-S composite synthesized *via* the interfacial emulsification method and the PANI-S composite synthesized *via* the SDS/HCl template method were separately mixed with a conductive agent (conductive graphite, Qingdao Tianyuan graphite Co., Ltd.) and a binder (polyvinylidene fluoride, PVDF, SOLVAY-SOLEF-9007) at the mass ratio of 8 : 1 : 1, respectively. Then, a small amount of deionized water and ethanol absolute (AR, Hubei xinrunde Chemical Co., Ltd) were added to the abovementioned mixture. The mixture was water-bath heated for 8 hours to obtain a uniform viscous slurry. The slurry was uniformly applied to an aluminum foil current collector with an applicator blade. After being dried at 60 °C in a vacuum drying oven (DZF 2001 type, Shanghai Yiheng Instrument Co., Ltd.) for 24 hours, the current collector was punched into circular electrode pieces with a diameter of about 1 cm. Using these punched pieces as a positive electrode, a lithium piece (16 × 0.6 mm 15.6 × 0.45 mm, Jiangsu Taizhou) as a negative electrode, the Celgard 2400 microporous film as a separator, and 1 mol L^−1^ of LiTFSI (DOL/DME, volume ratio of 1 : 1) as an electrolyte, button batteries were assembled in a glove box (Lab 2000). After being left undisturbed overnight, the assembled batteries were electrochemically tested. Constant current charge and discharge testing was carried out using the LAND CT2001A Blue Test System (Wuhan). The specific capacity and current multiplying ratio of the composite positive electrode were calculated based on the mass of the active substance sulfur.

## 3. Results and discussion

### 3.1 Characterization of PANI-S (synthesized *via* the interfacial emulsification method)

**Fig. 1:**
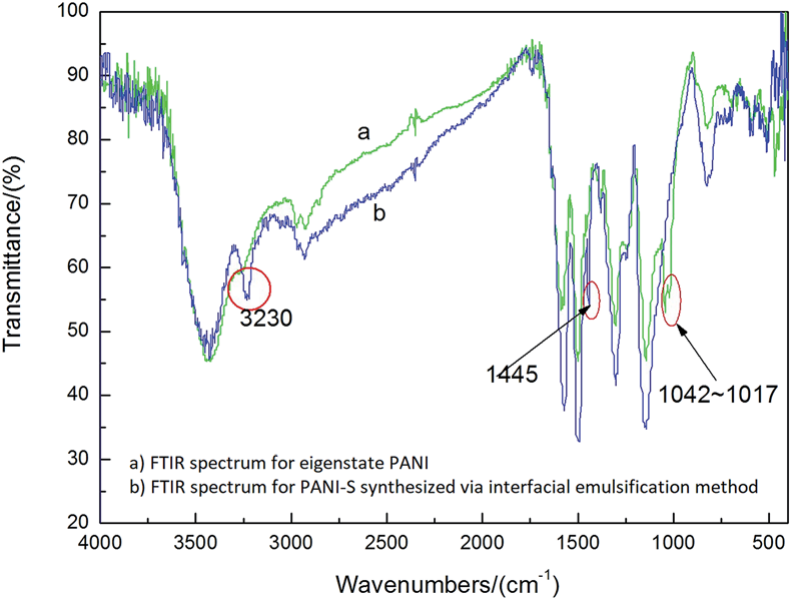
FTIR spectra of (a) eigenstate PANI and (b) PANI-S (synthesized *via* the interfacial emulsification method).

In the FTIR spectra of PANI-S (synthesized *via* the interfacial emulsification method) (Fig. 1), the signal at 1585 cm^−1^ was attributed to the vibration of quinoid N=Q=N and the signal at 1500 cm^−1^ was attributed to the vibration of benzene N–B–N, indicating that PANI was successfully synthesized. Compared with the FTIR spectra of eigenstate PANI, in the FTIR spectra of PANI-S (synthesized *via* the interfacial emulsification method), additional signals at 3230 cm^−1^ and 1445 cm^−1^ appeared, which were attributed to the association interaction between monomers and solvents;^19^ moreover, the signals between 1017 cm^−1^ and 1042 cm^−1^ were attributed to the effect of the dopant HCl.^20^

**Fig. 2:**
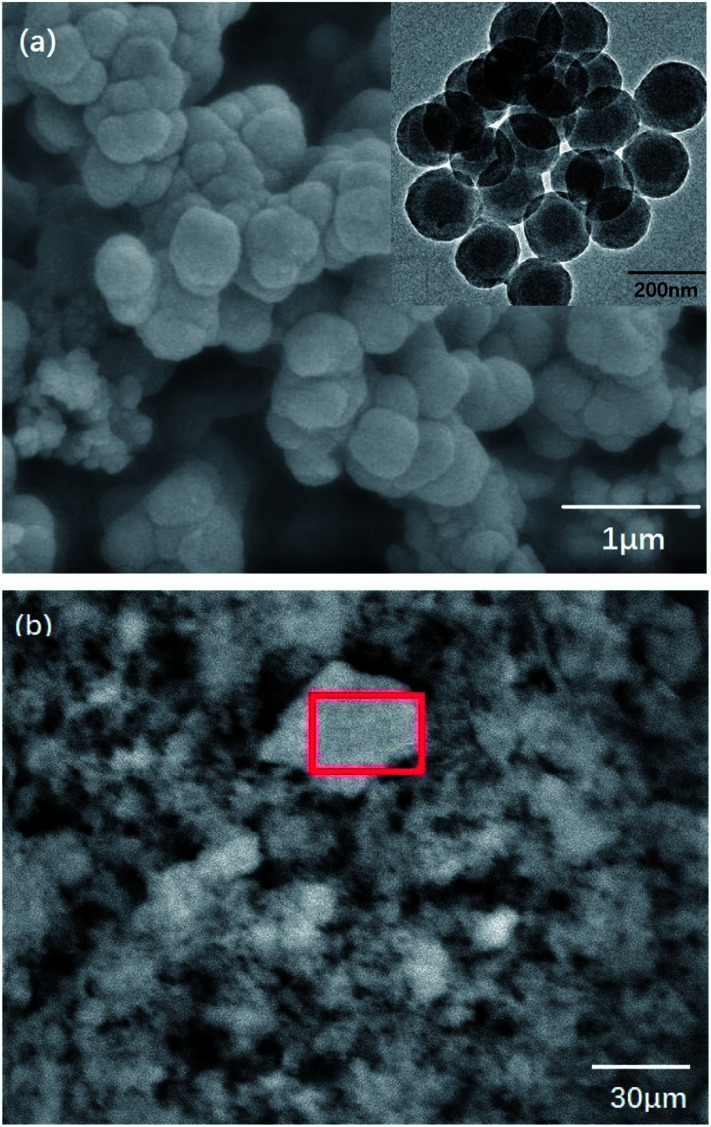
(a) SEM and TEM images and (b) floccule formation in PANI-S (synthesized *via* the interfacial emulsification method).

Triton-X100 is a kind of nonionic surfactant that is soluble in water. It can be used as an emulsifier in the inverse emulsion polymerization.^21^ During synthesis, the aniline monomer, oxidant APS, and dopant HCl diffused to the interface and began to polymerize. Originally, the product was polyaniline nanofibers whose hydrophilicity was greater than their lipophilicity. Thus, the polyaniline nanofibers moved to the water phase and entered the Triton-X100 micelle. Because of nucleation, a second growth process occurred and generated polymer latex particles^22–24^ that further coated the element sulfur in the system. In Fig. 2a, it can be clearly observed that the products have nano-microsphere morphology. Because the density of the organic phase is larger, the water phase remains on the upper layer of the organic phase. Because of Triton-X100 and gravity, the diffusion of nanofibers was inhibited; thus, the formation of nanotube PANI-S was impeded. However, several short PANI-S nanotubes were still generated. As shown in Fig. 2b, piles of floccule appeared obviously.

**Fig. 3:**
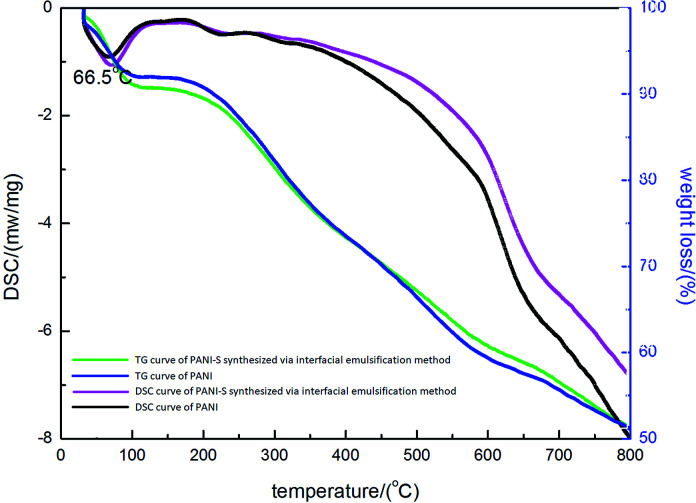
The TGA and DSC curves of PANI-S (synthesized *via* the interfacial emulsification method) and the contrast TGA and DSC curves of PANI.

To further demonstrate the elemental composition of the product, EDS was utilized to investigate the sulfur content. As presented in Table 1, the sulfur content is about 7 wt%. In Fig. 3, the weight loss curve of PANI-S is plotted against the reference curve of PANI. The decomposition step starting at 160 °C was attributed to the loss of sulfur, with a weight loss of up to 8 wt%, in accordance with the data obtained from EDS. Moreover, the DSC curve of PANI-S agreed with that of PANI; this showed that the addition of sulfur did not significantly change the thermal properties of PANI.

**Table 1:** EDS elemental analysis of nano-microsphere PANI-S

Element	Weight percentage	Atomic percentage
C K	71.39	77.79
N K	13.84	12.93
O K	7.92	6.48
S K	6.85	2.79

**Fig. 4:**
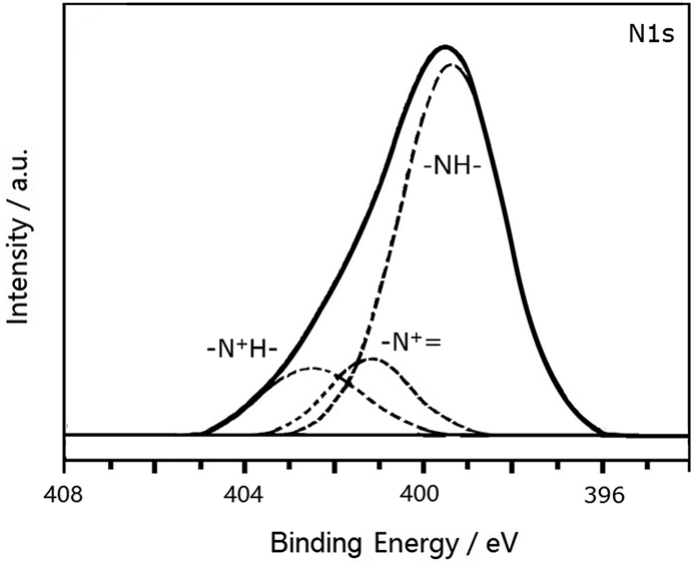
The N1s XPS spectrum of PANI-S (synthesized *via* the interfacial emulsification method).

Fig. 4 shows the N1s XPS spectrum of PANI-S (synthesized *via* the interfacial emulsification method). It can be observed that there are three peaks in the spectrum. The center of one peak appeared at 399.2 eV, which was attributed to –NH. Moreover, the other two peaks at the binding energies of 401.0 eV and 402.3 eV were attributed to protonated imine (–N^+^=) and amino nitrogen atom (–N^+^H–), respectively. The peak of quinoneimine (–N=) disappeared because HCl was utilized as a dopant and proton doping preferentially occurred on the nitrogen atom of quinoneimine.^25^ In addition, no S2p XPS spectrum was obtained for PANI-S (synthesized *via* the interfacial emulsification method); this proved that there was no sulfur on the surface of this composite.

### 3.2 Characterization of nanotube PANI-S (synthesized *via* the SDS/HCl template method)

**Fig. 5:**
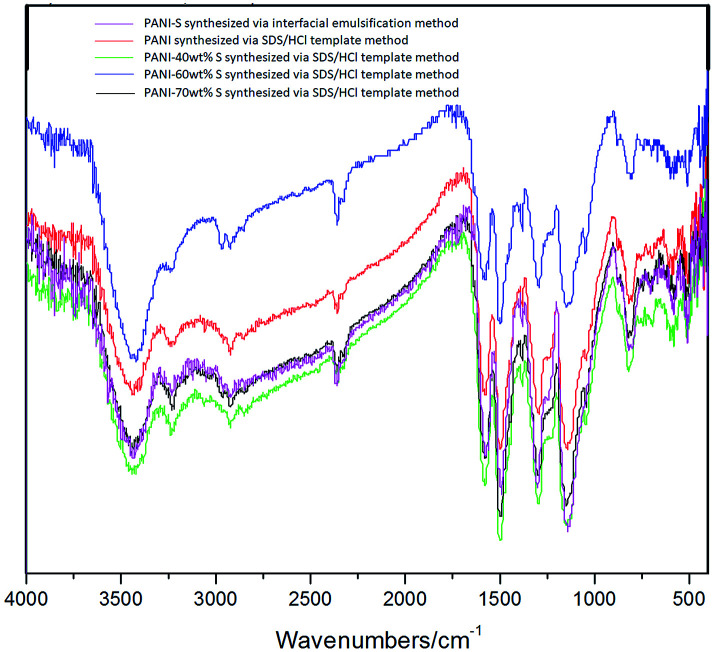
Infrared spectra of different kinds of PANI-S composites.

Fig. 5 clearly shows a comparison between the IR spectra of PANI-S (synthesized *via* the interfacial emulsification method), PANI (synthesized *via* the SDS/HCl template method), PANI-40 wt% S (synthesized *via* the SDS/HCl template method), PANI-60 wt%S (synthesized *via* the SDS/HCl template method), and PANI-70 wt% S (synthesized *via* the SDS/HCl template method). The signals in the range of the infrared spectrum fingerprint area (2000–800 cm^−1^) resembled each other. The signals at 1558 cm^−1^ and 1483 cm^−1^ were attributed to the vibration of quinoid N=Q=N and benzene N–B–N, respectively. The signals at 1305 cm^−1^ and 1245 cm^−1^ were attributed to the vibration of C–N in the quinone ring or benzene ring. The signals in the range of 500 cm^−1^–1100 cm^−1^ were attributed to protonic acid, which had been doped into the PANI molecular chains.^26–28^

During the synthesis of PANI *via* the SDS/HCl template method, SDS acted as a micelle template, providing “cavity” in a dynamic equilibrium; therefore, the aniline monomer and dopant could diffuse through the “cavity” wall.^29^ Under the action of HCl, the aniline monomers assembled along the SDS micelle, generating a nano polymer with a certain morphology. This meant that the nanostructure could be designed by changing the reaction conditions such as monomer concentration. Through experimental comparison, it was found that the main factors affecting the morphology of PANI were as follows: (1) monomer concentration; (2) SDS dosage; and (3) agitation, as presented in Table 2.

**Table 2:** Different reaction conditions for the synthesis of PANI *via* the SDS/HCl template method

Number	SDS (mg)	HCl (mL)	Aniline (mL)	APS (g)	H_2_O (mL)
1 (agitation)	65.3	0.5	1	2.32	350
2 (no agitation)	64.8	0.5	1	2.32	350
3 (no agitation)	100.4	1	2	4.63	350
4 (no agitation)	101.1	2	2	4.63	350
5 (no agitation)	153.8	2	4	9.26	350
6 (no agitation)	102.5	1.5	4	10.0	350

**Fig. 6:**
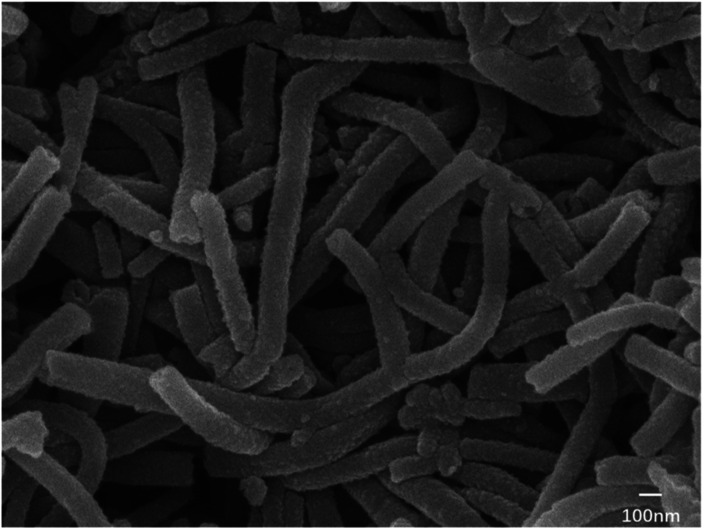
Electron microscopy image of nanotube PANI (synthesized *via* the SDS/HCl template method).

The result showed that nanotubes with a uniform thickness and regular shape (as shown in Fig. 6) could be generated under the following conditions: (1) the aniline monomer concentration of around 0.12 mol L^−1^ was selected because if the aniline monomer concentration was extremely low, the intermolecular force among assembled monomers would become significantly weak, which would affect the formation of nanotubes. (2) The SDS dosage of no less than 1 wt% was selected because if the SDS dosage was less than 1 wt%, no micelles would not be generated, and then, there would be no template for nanotube formation. (3) No agitation: the agitation would directly break the growth of the micelle and then disrupt the formation of nanotubes. Thus, static polymerization is the best way for nanotube growth.

**Fig. 7:**
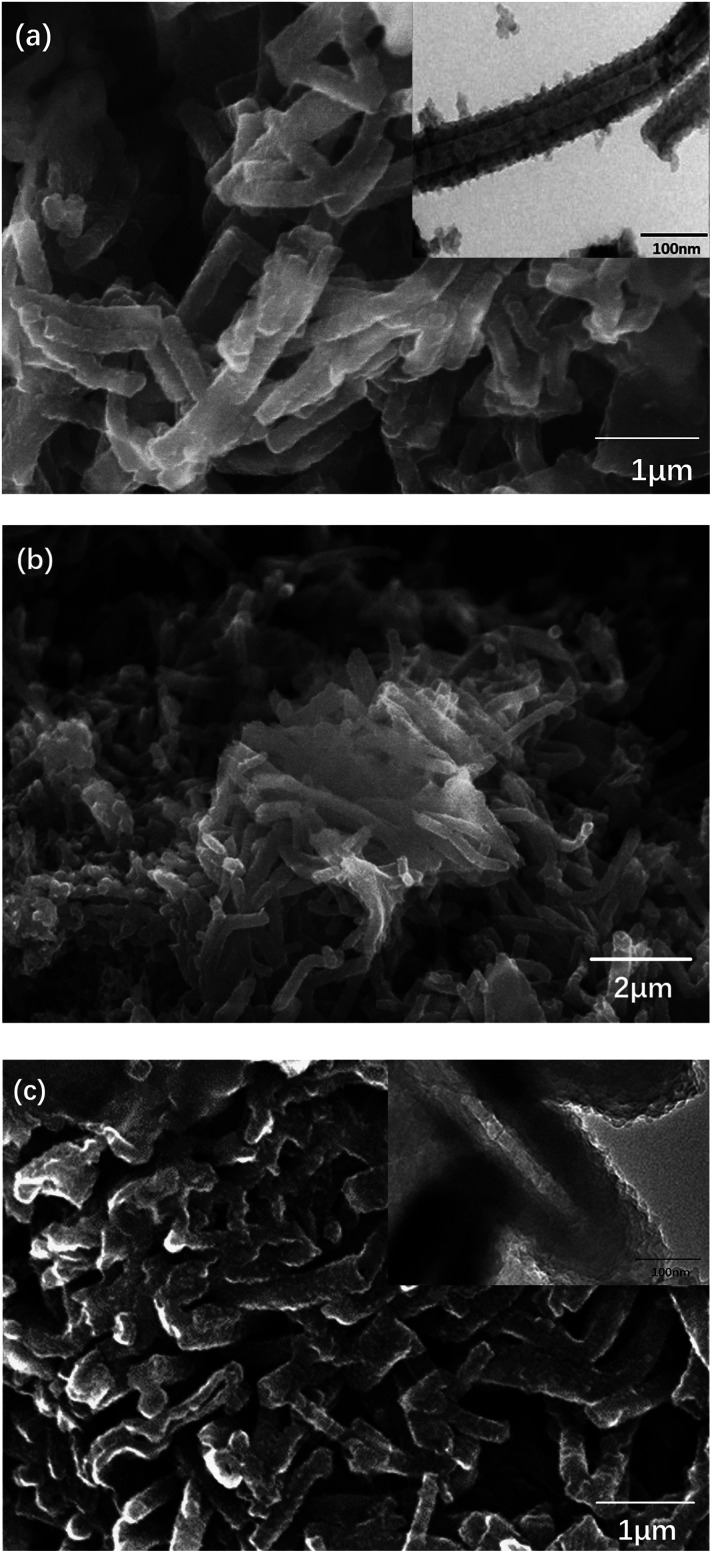
(a) SEM and TEM images of PANI (synthesized *via* the SDS/HCl template method) heated at 160 °C for 24 hours; (b) SEM image of PANI-40 wt% S (synthesized *via* the SDS/HCl template method) heated at 160 °C for 24 hours; and (c) SEM and TEM images of PANI-70 wt% S (synthesized *via* the SDS/HCl template method) heated at 160 °C for 24 hours.

Then, nanotube PANI was mixed and heated with sulfur (0, 40 wt%, and 70 wt%) at 160 °C for 24 hours. Heated PANI without sulfur had a diameter of about 70 nm (between 50 and 100 nm) and a length of about 1 μm; however, the morphology of the product barely changed, which can be observed in Fig. 7a, when compared with that of nanotube PANI, as shown in Fig. 6. However, when the sulfur content was increased to 40 wt%, the distribution of the product became non-uniform. It can be observed from Fig. 7b that the polymer began to accumulate because in the process of physical absorption, melted sulfur accumulated locally and bonded the PANI nanotubes together. When the sulfur content was increased to 70 wt%, nanotubes with a diameter of less than 100 μm disappeared basically (as shown in Fig. 7c); this was attributed to the further accumulation of the product.

**Fig. 8:**
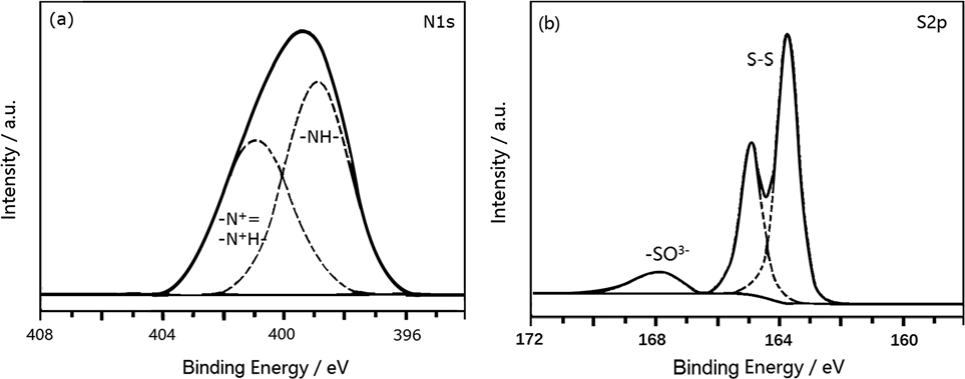
(a) The N1s XPS spectrum of PANI-S (synthesized *via* the SDS/HCl template method) and (b) the S2p XPS spectrum of PANI-S (synthesized *via* the SDS/HCl template method).

Fig. 8a shows the N1s XPS spectrum of PANI-S (synthesized *via* the SDS/HCl template method). There were two peaks in the spectrum. The center of one peak appeared at the binding energy of 399.2 eV, which was attributed to the nitrogen atom in –NH; the other peak appeared at the binding energy of 401.5 eV, which was attributed to the nitrogen atoms in both protonated imine (–N^+^=) and amino nitrogen atom (–N^+^H–).^25^ This PANI-S composite had an S2p XPS spectrum (as shown in Fig. 8b), which proved the existence of sulfur on the surface of this composite. Moreover, the typical double peaks appeared at 163.7 eV and 164.5 eV, which were attributed to the split peaks of S2p_3/2_ and S2p_1/2_, respectively. In addition, there was another peak at 168 eV, which was attributed to –SO^3−^ as CH_3_(CH_2_)_11_OSO^3−^ of SDS combined with the positively-charged polyaniline chain.

### 3.3 Electrical measurement

PANI-S (synthesized *via* the interfacial emulsification method), PANI-40 wt% S (synthesized *via* the SDS/HCl template method), and PANI-70 wt% S (synthesized *via* the SDS/HCl template method) were successfully employed as active cathode materials in a Li–S battery.

**Fig. 9:**
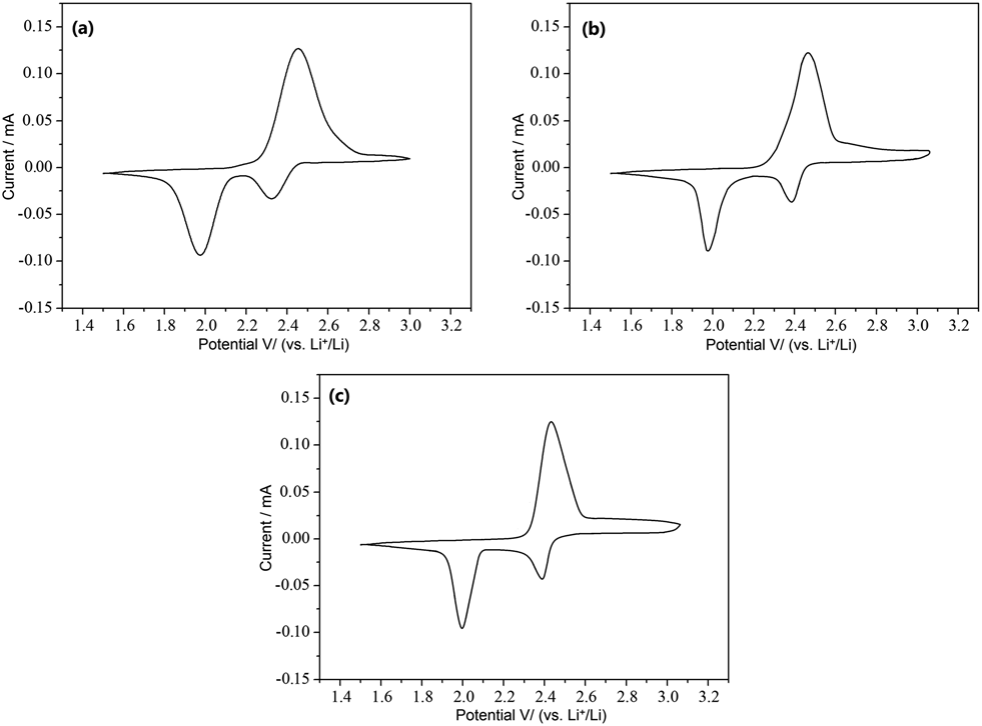
(a) The CV curves obtained after the first cycle of the nano-microsphere PANI-S (synthesized *via* the interfacial emulsification method) electrode; (b) the CV curves obtained after the first cycle of the nanotube PANI-70 wt% S (synthesized *via* the SDS/HCl template method) electrode; and (c) the CV curves obtained after the first cycle of the nanotube PANI-40 wt% S (synthesized *via* the SDS/HCl template method) electrode.

**Fig. 10:**
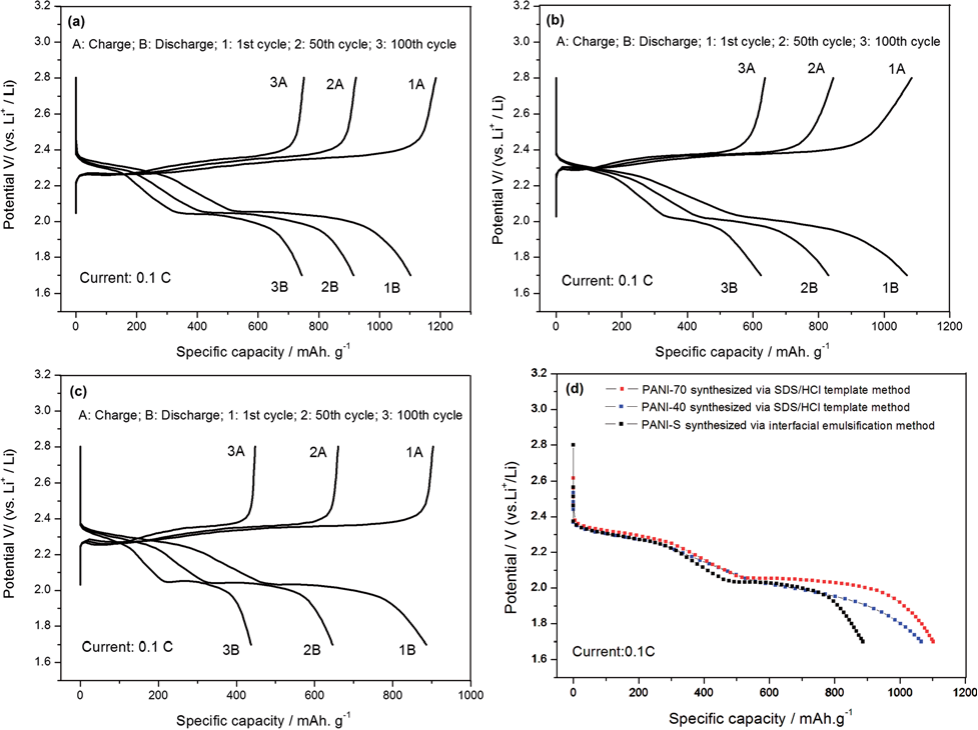
(a) The charge–discharge curves of the nanotube PANI-70 wt% S (synthesized *via* the SDS/HCl template method) electrode; (b) the charge–discharge curves of the PANI-40 wt% S (synthesized *via* the SDS/HCl template method) electrode; (c) the charge–discharge curves of the PANI-S (synthesized *via* the interfacial emulsification method) electrode; and (d) a comparison of the charge–discharge curves obtained after the first cycle of the three PANI-S composite electrodes.

Fig. 9 depicts the cyclic voltammetry (CV) curves obtained after the first cycle of the nano-microsphere PANI-S (synthesized *via* the interfacial emulsification method) electrode, nanotube PANI-70 wt% S (synthesized *via* the SDS/HCl template method) electrode, and nanotube PANI-40 wt% S (synthesized *via* the SDS/HCl template method) electrode in a 1 M LiTFSI/DOL-DME electrolyte at the scan rate of 0.05 mV s^−1^ and in the voltage range of 1.2–3.0 V. The three curves appeared nearly the same with two distinct reduction peaks mostly at 2.4 V and 2.0 V and one oxidation peak at 2.5 V, corresponding to the CV curve of element sulfur.^30^ At the open circuit voltage of 2.8 V and current of 0.1 C, all the charge–discharge curves of the three kinds of PANI-S composites exhibited two obvious plateaus (Fig. 10a–c) at 2.23 V and 1.90 V, characteristic for liquid electrolyte Li–S batteries. According to the study reported by Rauh and Yamin,^31,32^ the plateaus at 2.23 V and 1.90 V correspond to the conversion of elemental sulfur S_8_ to the high-valence polysulfide S_*x*_^2−^ (*x* = 4–6) and the further reduction of the high-valence polysulfide S_*x*_^2−^ to Li_2_S_2_, Li_2_S, *etc.*

**Fig. 11:**
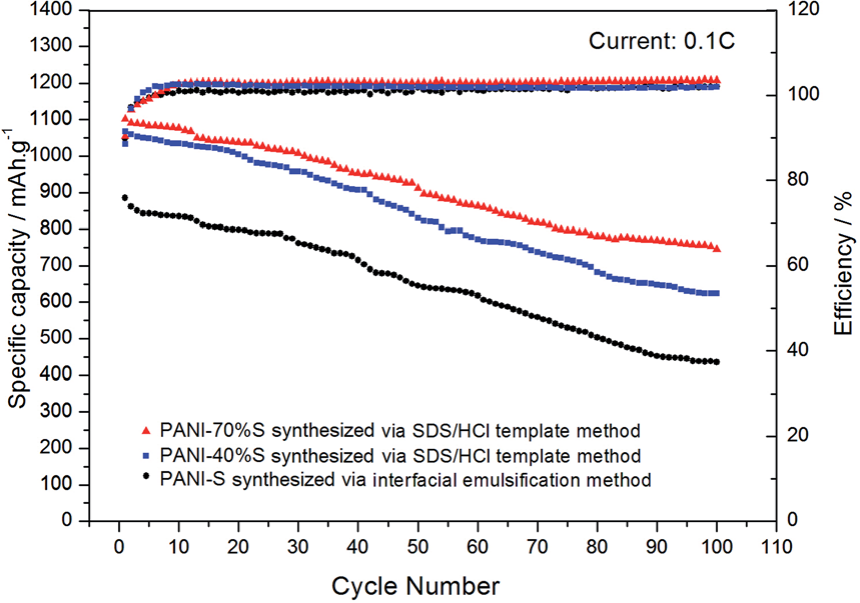
The cycle performance and coulombic efficiency curves of the PANI-S (synthesized *via* the interfacial emulsification method) electrode, PANI-40 wt% S (synthesized *via* the SDS/HCl template method) electrode, and PANI-70 wt% S (synthesized *via* the SDS/HCl template method) electrode.

As shown in Fig. 11, the initial specific capacity of the PANI-S (synthesized *via* the interfacial emulsification method) electrode was 885.2 mA h g^−1^. During the first 60 cycles of circulation, the specific capacity gradually decreased, and the 60th discharge specific capacity was about 618 mA h g^−1^. After this, the specific capacity gradually became stable until the 100th cycle and remained above 436 mA h g^−1^, with the efficiency of around 100% and capacity retention rate of around 49.3%. Comparatively, the initial specific capacity of the PANI-40 wt% S (synthesized *via* the SDS/HCl template method) electrode was 1068.25 mA h g^−1^ and the discharge specific capacity reduced to 772.8 mA h g^−1^ after 60 cycles of circulation and to 620 mA h g^−1^ after 100 cycles, with the efficiency of around 100% and capacity retention rate of around 58%; thus, the PANI-40 wt% S electrode showed a better cycle stability. The initial specific capacity of the PANI-70 wt% S (synthesized *via* the SDS/HCl template method) electrode increased to 1102.01 mA h g^−1^ and the discharge specific capacity could remain above 744 mA h g^−1^ after 100 cycles, with the efficiency of around 100% and capacity retention rate of around 67.5%; this electrode showed the best cycle stability of all the three kinds of electrodes. It was obvious that with an increase in the sulfur content, the proportion of the active substances increased and the specific capacity and cycle performance of the electrode improved. Comparatively, the electrochemical performance of the PANI-S (synthesized *via* the interfacial emulsification method) electrode was not so good. This was because during its synthesis, PANI connected with sulfur by electrostatic interactions, forming a coating structure.^33^ Although this structure could restrain the dissolution of polysulfide to some extent, during long cycling, nano-microsphere PANI could not always support the stress from volume deformation or retain the integrity of the outer part as the molecular force between the doped PANI particles was not significantly strong. Once the partially dissolved polysulfide diffused out of the coating, it would run to the lithium anode and would be reduced to irreversible lithium sulfide.^34^ Therefore, the binding effect of nano-microsphere PANI was limited to improve the cycle performance. However, nanotube PANI-S (synthesized *via* the SDS/HCl template method) had an interwoven network structure, which could effectively inhibit the dissolution of polysulfide. Moreover, its pores with diameter in the 7–100 nm range could absorb a small amount of polysulfide ion or polysulfide lithium, which was also conducive to reducing the residence of S_*x*_^2−^ in the electrolyte, to improve the performance of the lithium–sulfur battery.^35,36^

**Fig. 12:**
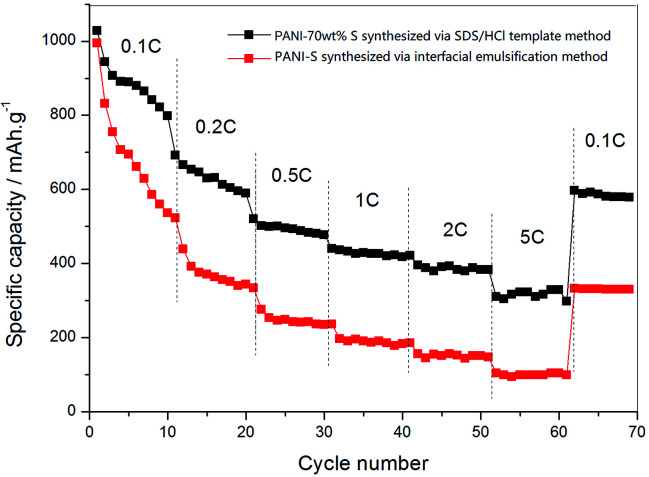
The rate performance curves of the PANI-S (synthesized *via* the interfacial emulsification method) electrode and PANI-S (synthesized *via* the SDS/HCl template method) electrode.

By gradually increasing the current density (0.1 C, 0.2 C, 0.5 C, 1 C, 2 C, and 5 C) during the charge–discharge process (10 times cycling at each current density), the rate performances of the PANI-S (synthesized *via* the interfacial emulsification method) electrode and PANI-S (synthesized *via* the SDS/HCl template method) electrode were obtained (Fig. 12). At different current densities, the discharge specific capacities of the PANI-S (synthesized *via* the SDS/HCl template method) electrode were about 885.31 mA h g^−1^, 523.18 mA h g^−1^, 334.04 mA h g^−1^, 236.15 mA h g^−1^, 186.03 mA h g^−1^, and 146.84 mA h g^−1^. When the current density was readjusted to 0.1 C, the discharge specific capacity of this electrode returned to 332.08 mA h g^−1^. Comparatively, the discharge specific capacities of the PANI-S (synthesized *via* the interfacial emulsification method) electrode were about 1088.20 mA h g^−1^, 691.80 mA h g^−1^, 520.65 mA h g^−1^, 440.02 mA h g^−1^, 421.33 mA h g^−1^, and 382.61 mA h g^−1^. When the current density was readjusted to 0.1 C, the discharge specific capacity of this electrode returned to 597.28 mA h g^−1^, suggesting a better rate performance. As nanotube PANI-S (synthesized *via* the SDS/HCl template method) had a larger specific surface area, it could contact more electrolyte. Moreover, its porous morphology was conducive to the infiltration of the electrolyte, resulting in a shorter diffusion path for lithium ions, which was conducive to the insertion and removal of lithium ions at high current densities;^37^ thus, this electrode had a better multiplier performance.

## 4. Conclusions

In this study, nano-microsphere PANI-S was synthesized *via* the interfacial emulsification method and nanotube PANI-S was synthesized *via* the micelle template method. These two kinds of products were characterized and prepared into electrodes. The employment of nanotube PANI-S (synthesized *via* the SDS/HCl template method) as a cathode material in Li–S batteries resulted in the higher initial specific capacities of up to 1102.01 mA h g^−1^, better capacity retention abilities (744 mA h g^−1^ after 100 cycles), and better rate performance; this suggests that excellent lithium–sulfur batteries can be prepared by designing an electrode material structure using nanotube PANI-S.

## Author contributions

The manuscript was written through the contributions of all authors.

## Funding sources

This work was supported by the National Natural Science Foundation of China (51774017)

## Conflicts of interest

There are no conflicts to declare.

## Supplementary Material
